# Unilateral Vocal Cord Paresis During Sleep Endoscopy

**DOI:** 10.7759/cureus.15157

**Published:** 2021-05-21

**Authors:** Francesca C Viola, Nicole M Favre, Matthew Kabalan, Michele M Carr

**Affiliations:** 1 Otolaryngology, Jacobs School of Medicine and Biomedical Sciences, University at Buffalo, Buffalo, USA

**Keywords:** obstructive sleep apnea, pediatrics, vocal cord immobility, drug-induced sleep endoscopy, unilateral vocal cord paresis

## Abstract

Abnormalities of vocal cord motion in children with obstructive sleep apnea (OSA) who undergo drug-induced sleep endoscopy (DISE) are not frequently described. A 17-year-old female with a history of asthma, reflux, and bipolar disorder had a history of poor sleep. Polysomnography (PSG) showed apnea-hypopnea index (AHI) of 13.9/hr, obstructive AHI 10.3/hr, and oxygen saturation nadir 87%. Physical exam showed BMI 34 and 3+ tonsils. She underwent DISE with propofol infusion, which showed partial obstruction at the palatine and lingual tonsil levels, a posteriorly displaced epiglottis along with immobility of the left vocal cord. Tonsillectomy was performed as planned. At her post-op visit, laryngoscopy showed normal vocal cord motion bilaterally. Post-operative PSG was improved.

## Introduction

Abnormalities of vocal cord motion in children with obstructive sleep apnea (OSA) who undergo drug-induced sleep endoscopy (DISE) are not frequently described. Unilateral vocal cord immobility may be due to neurologic, mechanical, iatrogenic, or idiopathic pathologies [1]. Studies have reported instances of unilateral vocal cord immobility after endotracheal intubation under general anesthesia [1,2]. Central nervous system pathology is another common cause [3]. Interestingly, the prognosis of unilateral vocal cord immobility is highly variable, ranging from persistent immobility to complete resolution [4]. The following case describes a teenage girl with a history of obesity, asthma, reflux, and bipolar disorder who was observed to have unilateral vocal cord immobility during DISE with normal vocal cord movement while awake.

## Case presentation

A 17-year-old female with a past medical history of gastroesophageal reflux disease, factor V Leiden, asthma, migraines, bipolar I disorder, depression, and anxiety presented with poor sleep. The patient reported daytime somnolence and difficulty falling asleep, although she reported being able to maintain sleep after sleep onset. The patient denied snoring. Her mother had severe OSA controlled with continuous positive airway pressure therapy. Physical exam was unremarkable apart from 3+ tonsils and a BMI of 34.6. No voice abnormalities were noted.

The patient underwent polysomnography, which recorded an apnea-hypopnea index (AHI) of 13.9 and an oxygen saturation nadir of 86.8%, consistent with OSA. There was no evidence of central apnea events. DISE with tonsillectomy and possible revision adenoidectomy were recommended and scheduled.

On the day of surgery, the patient was brought into the operating room and placed in a supine position for sleep endoscopy. Anesthesia was induced with inhaled anesthetics, an IV was placed, and a propofol drip was started and titrated. The inhaled anesthetic was stopped. There was no instrumentation of the airway prior to sleep endoscopy, specifically, there was no rigid laryngoscope placed and no intubation. Once the patient was snoring, a 4-mm flexible laryngoscope was used to inspect both nasal cavities, and then the scope was advanced to include examination of the nasopharynx, oropharynx, retrolingual airway, supraglottis, and glottis in the usual fashion for sleep endoscopy. The nasal airways were patent at the anterior inferior turbinate with only mild inferior turbinate hypertrophy. There was no significant adenoid tissue present. On examination of the soft palate, the airway was noted to narrow in the anterior-posterior direction. Palatine tonsils obstructed about half of the airway. The lingual tonsil was found to be enlarged. The epiglottis was posteriorly displaced and made two-point contact with the posterior pharyngeal wall. With chin lift, the aryepiglottic folds demonstrated mild shortening with a partially retracting epiglottis. The vocal cords were visualized next, and there was left vocal cord immobility, which can be seen in Video 1.

**Video 1:** Sleep endoscopy showing left vocal cord immobility

There was no visible injury or lesion of the left vocal cord. The right vocal cord demonstrated relatively normal mobility. The scope was removed and the patient was intubated and positioned for tonsillectomy, which was uneventful. After the procedure, the patient was awakened and extubated without any complications. There was no post-operative bleeding or dehydration.

The patient was seen for her postoperative follow up two months later. The patient was informed of the left vocal cord immobility seen during sleep endoscopy. The patient denied any previous diagnosis of vocal cord abnormalities and denied any history of hoarseness, voice changes, or aspiration. She had a history of speech therapy, but the family was unable to provide further details about the indications.

At this postoperative follow-up visit, the patient underwent flexible laryngoscopy in the office, while awake. The patient was in a seated position on the exam chair. The nasal cavities were anesthetized and decongested using a combination of tetracaine and oxymetazoline. During this awake endoscopic examination, the nasal cavities were found to be unremarkable aside from some mild inferior turbinate hypertrophy, the nasopharynx was open, the oropharynx was unremarkable, and the supraglottis was also found to be normal in appearance. Findings are shown in Video 2. In contrast to her findings on sleep endoscopy, the vocal cords were found to be bilaterally and symmetrically mobile. Overall, on this examination, the appearance of the larynx was entirely normal. The patient reported an uncomplicated recovery from surgery and stated that she had returned to her normal activities and diet. A two-month postoperative polysomnogram demonstrated an AHI of 3.6/hr and an oxygen saturation nadir of 92%.

**Video 2:** Flexible laryngoscopy while awake

## Discussion

Unilateral vocal cord immobility is an uncommon type of vocal cord dysfunction. Most causes are related to neurologic, mechanical, iatrogenic, or idiopathic pathologies [1]. For example, studies have reported instances of unilateral vocal cord immobility after endotracheal intubation while under general anesthesia [1,2]. It is postulated that this is due to cricoarytenoid joint damage during endotracheal tube insertion. The endotracheal tube can also compress the recurrent laryngeal nerve, especially in prolonged procedures. Central nervous system pathology, such as multiple system atrophy, has also been reported to be associated with vocal cord paresis [3]. Other studies have also observed vocal cord abnormalities during DISE in children, specifically paradoxical vocal cord dysfunction and vocal cord abductor paralysis [3,4]. Although Newby et al. [4] reported a different type of vocal cord abnormality from our patient, it does demonstrate that vocal cord movement abnormality can be manifested during sleep endoscopy. This raises questions about whether these anomalies occur during sleep or whether DISE with concomitant medication administration plays a role in these presentations. Unfortunately, there is no literature claiming that anesthetic agents are associated with unilateral vocal cord immobility so we will not speculate about the role of anesthetic agents in this case. Given the variety of contributors to vocal cord paralysis, the prognosis of unilateral vocal cord paralysis is highly variable, ranging from little improvement to full resolution [5].

The presence of unilateral vocal cord paralysis only during DISE, with complete resolution while awake, is previously unreported. The presence of other comorbidities further complicates the clinical picture. This raises questions about the mechanism of unilateral cord paralysis, what clinical significance it may have, and possible connections to psychiatric, neurologic, and pulmonary comorbidities.

One theory regarding the mechanism of unilateral vocal cord paralysis in this individual is the unmasking of underlying neuromotor insufficiency while under anesthesia. Although the current literature on this mechanism is sparse, it is possible that deepening anesthesia could result in the emergence of unilateral vocal cord weakness that is normally compensated for when the individual is awake. This mechanism can be conceptualized similarly to the well-described concept of aging, which leads to a reduction in functional skeletal muscle mass and a loss of skeletal muscle performance that can decrease the ability to compensate for underlying neuromotor insufficiency [6]. While our patient was asymptomatic when awake and muscle dysfunction may be sufficiently compensated for, the use of general anesthetics, such as propofol, increases the inhibitory tone of the central nervous system resulting in decreased neuromuscular compensation [7].

Though the cause and importance of vocal cord dysfunction during sleep are unknown, physicians should be vigilant to document instances of vocal cord immobility during DISE. Currently, there is no universally accepted method of DISE evaluation and scoring [4]. Tejan et al. [8] looked at multiple DISE scoring systems and found that each of them emphasized different anatomic sites, employed different numerical scales to quantify obstruction, and had unique methods to characterize the airway. In an attempt to standardize DISE interpretation, the VOTE classification was developed [9]. Despite improvements in reporting criteria, many DISE scoring systems that have been published do not include vocal cord movement, further complicating the documentation of vocal cord anomalies during sleep [10]. Despite these drawbacks, sleep surgeons should be cognizant of vocal cord movement during sleep endoscopy.

## Conclusions

This is the first report in the literature describing a unilateral vocal cord movement abnormality during sleep that was not present while awake. The OSA resolved after tonsillectomy and her awake laryngeal function has remained normal at her six-month follow-up. The significance of this finding is unknown, so we plan on close monitoring of the patient. Surgeons should be vigilant in the visualization and assessment of the vocal cords during DISE and record a detailed account of their findings.
